# Relationship between parent and child pedometer-determined physical activity: a sub-study of the CANPLAY surveillance study

**DOI:** 10.1186/1479-5868-10-8

**Published:** 2013-01-18

**Authors:** Cora L Craig, Christine Cameron, Catrine Tudor-Locke

**Affiliations:** 1 Canadian Fitness and Lifestyle Research Institute, Ottawa, ON K2P 0J2, Canada; 2 School of Public Health, University of Sydney, Sydney, Australia; 3 Walking Behaviour Laboratory, Pennington Biomedical Research Center, Baton Rouge, LA 70808, USA

## Abstract

**Background:**

Understanding parental influences on their children’s physical activity (PA) provides insight into developing effective family-based interventions. This study examines whether children’s objectively monitored PA is associated with that of their parents.

**Methods:**

Participants (children and parents) were recruited to a sub-study of the Canadian Fitness and Lifestyle Research Institute’s annual Canadian Physical Activity Levels among Youth (CANPLAY) surveillance study. In total, 539 of 1,187 eligible children (age range 5–19 years) and at least one of their parents participated. Participants logged pedometer steps for 7 days. Descriptive statistics were used to examine parental mean daily steps by their children’s age, sex and steps/day. Associations between steps/day for parents and children (controlling for their age and sex) were estimated using general linear and logistic regression.

**Results:**

Children’s mean steps/day did not differ by parents’ age or sex, nor by whether one or both parents participated in the study. There were quantifiable relationships between parents’ and children’s steps/day. For every 1,000 step increase in a father’s steps/day, his son’s increased by 329–407 steps/day and his daughter’s increased by 273 steps/day (adjusted model only). Every 1,000 step increase in a mother’s steps/day was associated with 263–439 extra steps/day for her son’s steps/day and 195–219 steps/day for her daughter. A 3,000 step increment in a father’s or mother’s steps/day was associated with 1.9-2.5 fold increase in the odds that their child’s activity level would be in the upper two tertiles of steps/day.

**Conclusions:**

These cross-sectional data indicate that children’s PA is related to that of their parents in distinct and quantifiable ways. Interventions are warranted to evaluate the direction of this relationship, confirm the magnitude of influence, and illuminate mediating and moderating mechanisms by which both parents may have influence over their own children’s PA.

## Background

As an important health-promoting behaviour, children’s physical activity (PA) is logically shaped, at least to some extent, by a combination of genetic and environmental influences supplied by their parents. This phenomenon is typically known as familial aggregation [1] or clustering [2] of shared risk factors, including health behaviours such as PA. Earlier studies of familial similarities in PA behaviour were limited primarily to subjective estimates of recalled time spent in demanding leisure time activities [3]. Advances in body worn technologies enabled more precise comparison of objectively detected movement in children and parents. Specifically, Freedson et al. [4] demonstrated that evidence of familial aggregation of PA was present in 67% of father-child dyads and 73% of mother-child dyads based on Caltrac activity counts [4], whereas Loucaides and Jago found no evidence of familial correlations in step counts using a Yamax DW 200 Digiwalker [5]. Using a more sophisticated accelerometer, Jago and colleagues [6] reported no associations between time that parents and children spent in moderate-to-vigorous PA (MVPA). However, this finding may only reflect engagement primarily in volitional exercise and sport (i.e., higher intensity behaviours, which research shows relatively few people in a population actually engage in when assessed by accelerometer [7]), and may not capture the full spectrum of PA that includes habitual and lower intensity behaviours that may be more explainable by familial aggregation. Other accelerometer-based studies have shown parent–child associations in total PA [8-10]. More recently, researchers out of France documented correlations between mothers’ and their children’s pedometer-determined ambulatory PA (r = .21), and especially between mothers and daughters (r = .24), but almost no relationships evident between fathers and their children [11]. The rationale for continuing to examine PA behaviours captured by pedometers is that this lower-technology instrument is less expensive than research accelerometers and therefore more accessible to a range of practitioners and to parents and children themselves. Any findings are therefore more readily translatable to both clinical and real-life applications.

Understanding parental influences on their children’s PA provides insight into developing effective family-based interventions. The purpose of this study was to extend the limited understanding of the association between parent’s and children’s pedometer-determined PA, while attempting to provide a practical indication of the potential impact of parents’ behaviour on their children’s behaviour.

## Methods

### Data collection

Participants were recruited as a sub-sample within the Canadian Fitness and Lifestyle Research Institute’s annual Canadian Physical Activity Levels among Youth (CANPLAY) surveillance study. Details of CANPLAY have been published elsewhere [12]. In brief, CANPLAY participants are recruited by the Institute for Social Research (ISR) at York University via random digit dialling of a random sample of households representative of the population and stratified by province and territory. Within households, a respondent 20 years of age or older who was identified as a parent or legal guardian of a child between 5 and 19 years of age living in the household was selected and informed of the purpose of the study. Unlike the main CANPLAY study, which only recruits children into the study, this subsample also recruited their parents. Parent’s gender and age, household income (<$60,000, $60,000-$100,000, and ≥ $100,000) and child’s sex, age (in years), height (imperial or metric as preferred), weight (pounds or kilograms) and time spent watching television between school and dinner on a typical school day (a timeframe consistently related to physical inactivity in youth [13]) were asked during the recruitment interview. In total, 620 families with 1,187 children were recruited into the study. Of the 539 children (46%) who provided step data, steps/day for at least one parent was available for 256 boys and 283 girls. No significant difference was observed between participants and non-participants by responding parent’s age, sex, self-reported PA level, or by children’s age, sex, weight status, or time spent watching television; however, a higher proportion of participants (42%, 95% CI 33-52%) than non-participants (25%, 95% CI 18-33%) were from high income households.

Once recruited, the family was mailed a self-monitoring package that included: 1) a letter describing study and ethical contacts, 2) consent/assent form to be signed by parents/children over 14 years to participate in the study 3) SW-200 pedometers for each participating child and parent, 4) step log, 5) an illustrated and detailed guide describing how to wear the pedometer, 6) a letter for teachers and coaches about the study in case they questioned the child’s wearing of the pedometer during class or lessons, 7) a small gift of thanks, and, 8) a postage-paid reply envelope. Participating families received a short reminder telephone phone call a few days after receipt of the package, during which any questions were answered and participants were reminded to return the completed step log/assent form and pedometer at the end of their data collection. Participants were asked to wear the pedometer for 7 consecutive days and record daily steps on the provided log. A reminder letter was mailed within 6–8 weeks of original mailing to prompt completion of the study in the case of delayed response. An earlier study found no evidence of reactivity among children participating in CANPLAY [12]. Ethics approval was granted by the Ethics Review Board, York University.

### Data treatment and analysis

Step counts below 1,000 and above 30,000 steps/day were truncated to these values [14] and included in this analysis. Sex-specific tertiles of child’s steps/day were computed. Means and 95% Confidence Intervals (CI) were computed for sex- and age-specific steps/day (averaging steps taken over logged days) for parents and children. Means and CI for child’s steps/day were calculated for child’s age and sex, parent’s age and sex and parent’s steps/day by child’s age and sex. Weight status was determined by calculating body mass index as weight (kg)/height (m^2^) and categorizing into overweight and obese according to age- and sex-specific BMI cut-offs [15]. Descriptive statistics were computed using SPSS Complex Sample procedures (SPSS, Chicago, Illinois), which weighted the data to be representative of the population to account for the sample design and computed statistics based on the stratification and clustering inherent in the design. We regressed children’s steps/day on parent’s steps/day stratified by child’s sex with and without the inclusion of child’s age as a covariate. Similar patterns of results were obtained regardless of age adjustment, so the models without age controls are presented to maximize power. Given earlier findings that revealed an association between children’s steps/day, television viewing time and weight status [16], we then modelled the parent–child associations of steps/day adjusting for weight status and television viewing time as well as parent and child age and household income. Records with missing weight status, primarily due to missing height (33.2% of records), were excluded from the adjusted model. We also computed the odds of children being in the highest tertile of steps/day associated with 1,000 step incremental increases in parent’s steps/day and tested significant relationships using SPSS Complex Regression and Complex Logistic Regression procedures.

## Results

Roughly equal proportions of boys and girls participated in the study, with mean age 12.3 years and 11.4 years respectively (Table 1). There was no significant difference in mean parent’s age by sex of parent or child. In 45% of cases, pedometer data were available for the child and both parents. More mothers than fathers, particularly of boys (40% vs. 15%) participated when only one parent was involved in the study.

**Table 1:** Participant characteristics stratified by child’s sex

	**Boys (n = 256)**			**Girls (n = 283)**		
		**SD**	**95%CI**		**SD**	**95%CI**
**% of sample**	**48.7**	**3.2**	**(42.5, 54.9)**	**51.3**	**3.2**	**(45.1, 57.5)**
Child’s age (mean)	12.3	0.4	(11.6, 13.0)	11.4	0.5	(10.5, 12.3)
5 – 9 y (%)	27.0	3.5	(20.2, 35.1)	37.9	4.8	(28.4, 48.5)
10 – 14 y (%)	39.9	4.2	(31.6, 48.8)	38.3	4.4	(29.6, 47.8)
15 – 19 y (%)	33.1	4.3	(24.7, 42.6)	23.8	3.7	(16.5, 33.1)
Mother’s age (mean)	41.4	10.6	(40.1, 42.7)	40.1	11.2	(38.8, 41.4)
Father’s age (mean)	43.4	10.6	(42.1, 44.7)	42.6	13.7	(41.0, 44.2)
Parent’s participation						
mother only %	33.7	4.9	(24.1, 43.2)	39.3	5.6	(28.4, 50.3)
father only %	15.4	3.9	(7.8, 22.9)	19.8	4.7	(10.6, 28.9)
both parents %	51.0	5.3	(40.7, 61.2)	40.9	6.0	(29.1, 52.7)
Child’s steps/day (mean)	12,037	6,449	(11,247, 12,828)	10,587	4,987	(10,006, 11,168)
5 – 9 y	13,318	10,694	(12,008, 14,627)	11,390	7,562	(10,509, 12,272)
10 – 14 y	12,627	7,902	(11,659, 13,595)	10,781	7,107	(9,953, 11,609)
15 – 19 y	10,279	13,029	(8,683, 11,874)	8,999	11,029	(7,714, 10,284)
Mother’s steps/day (mean) by child’s age	8,411	6,441	(7,622, 9,200)	8,215	6,763	(7,427, 9,002)
5 – 9 y	8,732	8,686	(7,668, 9,796)	8,883	9,621	(7,762, 10,003)
10 – 14 y	8,248	8,065	(7,260, 9,237)	7,980	9,647	(6,856, 9,103)
15 – 19 y	8,351	12,678	(6,798, 9,904)	7,456	14,394	(5,779, 9,134)
Father’s steps/day (mean) by child’s age	9,090	7,478	(8,174, 10,006)	8,759	8,660	(7,750, 9,768)
5 – 9 y	8,313	7,951	(7,339, 9,288)	8,644	8,617	(7,640, 9,647)
10 – 14 y	9,594	11,094	(8,235, 10,953)	9,339	14,488	(7,651, 11,027)
15 – 19 y	9,183	15,273	(7,312, 11,054)	8,221	12,857	(6,723, 9,718)
Weight status						
healthy weight %	72.6	5.1	(62.6, 80.7)	73.5	5.7	(62.4, 82.2)
overweight%	19.7	3.4	(13.1, 28.5)	17.3	3.3	(10.9, 26.4)
obese %	7.7	1.9	(3.9, 14.7)	9.2	2.3	(4.6, 17.6)
Television watching after school (mean minutes)	42.8	62.9	(35.1, 50.5)	36.1	130.5	(20.9. 43.3)

**Figure 1:**
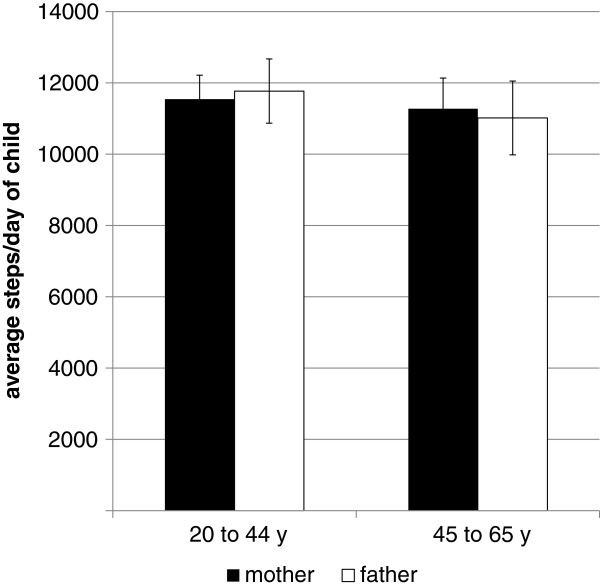
Child’s steps/day by parent’s age.

Boys took more steps/day than girls (mean delta =1,450) and step/day decreased by age group (from 13,318 to 10,279 for boys and from 11,390 to 8,999 for girls, between the ages of 5 and 19). Parents took fewer steps/day than children (mean delta =2,693). Parent’s steps/day did not differ by parent’s or child’s sex or age. Furthermore, children’s steps/day did not differ by the age or sex of their parent (Figure 1).

**Figure 2:**
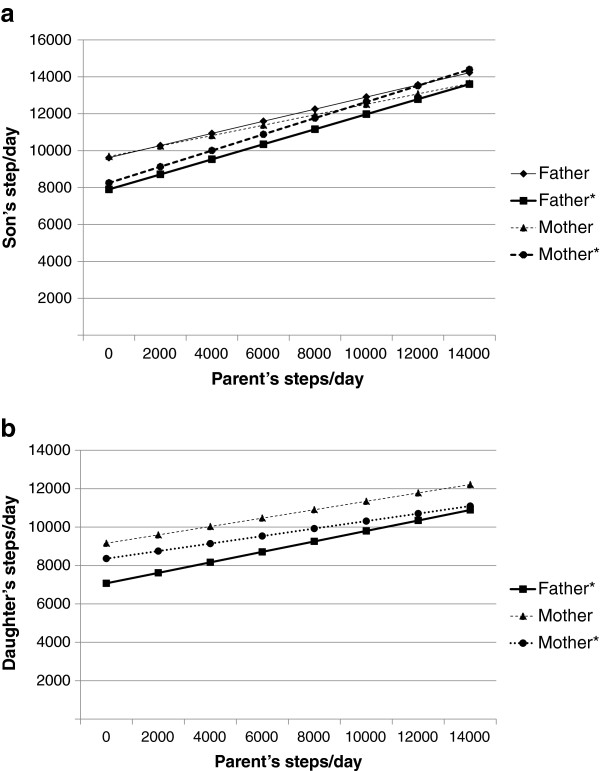
**a. Relationship between parents’ and childrens steps/day, boys.** *adjusted for parent and child age, household income, weight status and television viewing time. Unadjusted (Father, Mother) and adjusted models (Father* and Mother*) significant p < .05. **b**. Relationship between parents’ and childrens’s steps/day, girls. *adjusted for parent and child age, household income, weight status and television viewing time. Unadjusted model (Mother) significant p < .05; adjusted models (Father* and Mother*) significant p < .10.

Boys’ steps/day were related to their parents’ steps/day both before and after adjustment for parent and child age, household income, weight status and television viewing time (Figure 2a). Each 1,000 step increase in a father’s steps/day was associated with an unadjusted (p = 0.046) extra 329 daily steps and to an adjusted (p = .005) extra 407 daily steps in his son’s activity level. In the adjusted model, weight status was a significant modifier (p = .005) shifting the relationship by 702 fewer steps/day among overweight boys and 4,501 fewer steps among obese boys compared to those with healthy weights. Each 1,000 step increase in a mother’s steps/day was linked to an increase of 263 (unadjusted, p = 0.034) to 439 steps/day (adjusted, p = .004) for her son. Again, weight status was a significant modifier (p = .004) in the adjusted model, increasing steps/day by 1,840 among overweight boys and decreasing steps by 2,293 among obese boys. In contrast, girls’ steps/day were associated with their mothers’ steps/day in both adjusted and unadjusted models and only with their father’s steps/day after adjustment (Figure 2b). Each 1,000 step increase in a mother’s steps/day was associated with an increase of 195 (adjusted, p = .083) to 219 (unadjusted, p = .027) steps/day for her daughter. Each 1,000 step increase in a father’s steps/day was linked to an increase of 273 steps/day in the adjusted model only (p = .017, unadjusted model increase 105 steps/day, p = .343). Unexpectedly, each 30 minute increase in television viewing time reported for girls was linked to an increase of 387 steps/day (p = .025) controlling for weight status, father’s steps/day, father’s and daughter’s age, and household income.

**Figure 3:**
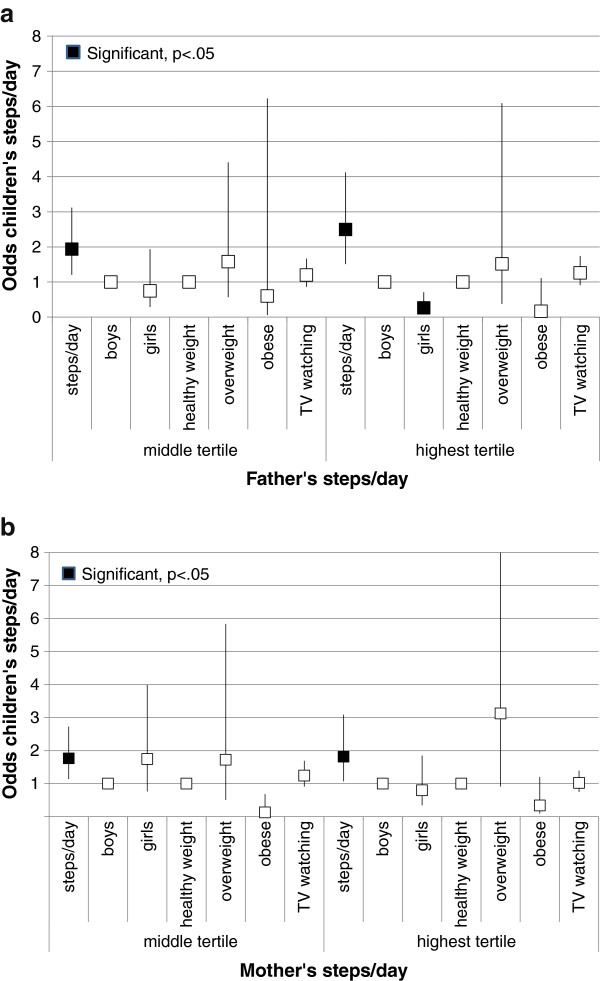
**a. Odds of children being in the two highest versus lowest tertile of steps/day by fathers’ steps/day.** Steps/day in 3000 step/day increments. TV watching time in 30 minute increments. **b**. Odds of children being in the two highest versus lowest tertile of steps/day by mothers’ steps/day. Steps/day in 3000 step/day increments. TV watching time in 30 minute increments.

Figures 3a and b plot the adjusted odds associated with the child’s chance of being in the middle and highest age-sex-adjusted tertile of steps/day by 3,000 steps/day increments in the parent’s steps/day (equivalent to 30 minutes of MVPA in adults [17]). A 3,000 step increment in a father’s steps/day was associated with 1.9-2.5 fold increase in the odds that their child’s activity level would be in the two highest tertiles of steps/day. However, being in the highest tertile was associated with being 30% less likely for girls, controlling for father’s steps/day. Similarly, an increase of 3,000 steps in a mother’s steps/day was associated with 1.8 times the likelihood that her child’s steps/day would be in the two highest age-adjusted tertiles of pedometer-determined PA.

## Discussion

Gustafson and Rhodes [18] concluded that there was no evidence of familial patterns of PA based on a systematic review of 24 studies; however, they noted that most of these studies did not employ objective or otherwise validated measures of PA. In contrast, the results of this CANPLAY study indicate a clear and quantifiable association between parent and child objectively monitored ambulatory PA levels. Although these are cross-sectional data, the findings suggest that efforts spent to increase PA levels among parents may also increase their children’s steps/day, and vice versa. This assertion, of course, requires a longitudinal or intervention design to confirm. Specifically, the relationship observed was such that if a father’s daily steps could be increased from the typical 8,759-9,090 step/day observed in this study to 10,000 steps/day [19] (e.g., as in the successful community-wide interventions to increase the proportion of adults taking 10,000 steps/day in Rockhampton, Australia [20] and Ghent, Belgium [21]), his child would be 1.25 times as likely to be in the middle tertile and 1.36 times as likely to be in the highest tertile of his or her peers’ daily steps. Furthermore, if a mother’s steps/day were increased to 10,000 steps/day from the observed mean of 8,215–8,411 steps/day, her child would be 1.21-1.22 times more likely to be in the two highest tertiles of their peer’s steps/day.

Fuemmeler et al. [10] used accelerometers to examine the relationship between parent–child activity levels, specifically time spent in MVPA, during specific time periods (weekends, weekday mornings before school and weekday afternoons between 3 and 7 pm). They found that parent’s MVPA was correlated with child’s MVPA irrespective of time period, and more specifically that mother’s and daughter’s MVPA were correlated in each of these time periods whereas father’s and daughter’s MVPA were correlated only weekday mornings. In contrast mother’s and son’s MVPA were not related and father’s MVPA was related to son’s MVPA on weekends and with weekday afternoons. These results are similar in part to these CANPLAY findings in that parent–child patterns of overall PA behaviour were related; however, mother-son results differed between the two studies. The largest differences between the two studies, however, is that Fuemmeler et al. [10] focused on the relationships at higher intensities of the PA (i.e., MVPA) accumulated within specific, possibly parent–child shared time frames, whereas CANPLAY examined parent–child relationships for total volume of PA (recorded as steps/day), and regardless of possible shared time with each other.

It has been suggested that parental modelling of PA is insufficient to influence child PA [22], but parent’s PA may be indicative of parental support of PA [23]. Based on their review of the extant literature, Gusto and Rhodes concluded that parental support such as encouragement, involvement and facilitation (transportation, equipment, access to opportunities to be active) may mediate any parent–child PA relationship. Bradley et al. [24] studied the impact of parenting processing, specifically monitoring and encouragement, and parent reported levels of their own physical activity, on changes in accelerometer-determined MVPA of children between 9 and 15 years of age. Each variable demonstrated significant but small effects that were sex-specific and moderated by age of puberty and region, so the mechanisms by which parents may influence their child’s PA may be multifactorial and not necessarily require their immediate presence at the time of the behaviour. These CANPLAY data suggest that relatively modest increases in parent’s PA may facilitate meaningful differences in their children’s PA. In this study, we found father-son and mother-son associations of steps/day with and without adjustment for potential confounders. This suggests that son’s PA behaviours may be influenced by parental modelling of physical activity. However, it may be that parents who are more active are also more likely to encourage and support their child’s activities and that it is this support rather than simply the modelling of behaviour that influences their child’s level of physical activity. The father-daughter and mother-daughter associations of steps/day were less robust. In the unadjusted model, only mother’s steps were associated with daughter’s steps. After adjustment for parent and child age, household income, weight status and television viewing time, mother’s steps/day were associated her daughter’s at the p < .10 level, but only father’s steps/day were associated with daughter’s steps/day at the p < .05 level and contrary to expectation, television viewing was associated with increased steps/day. This lack of consistency in the associations suggests that parents’ direct modelling of ambulatory behaviour may not be as important in shaping girls’ PAbehaviours as other factors such as competing interests (e.g, television viewing) and access to opportunities in higher income households. Further research is needed on moderating and mediating factors to illuminate potential mechanisms underlying successful family-based interventions designed to help parents increase their own PA as well as that of their children.

The CANPLAY data are cross-sectional and these associations, although significant, do not confirm causality. There may be other unmeasured factors that explain both parents’ and their children’s PA level that cannot be addressed simply by targeting parental PA behaviour. These findings are also based on a relatively small subsample of the CANPLAY data, specifically families who agreed collectively to self-monitor their behaviour for this survey. Parents who respond to this survey tended to be more active than their peers and have a university education, and children who responded were disproportionately representing the 5–10 age group [14], therefore the results of this analysis can only be generalized to those who share similar characteristics. Weight status and television viewing time are based on parental reports and suffer the problems inherent in self report data and likely reflect under-reporting of weight and television viewing time and over-reporting height as well as a large proportion of missing data for height which a large number of parents could not estimate. CANPLAY also uses pedometers to collect objective physical activity, and unlike accelerometers, these less expensive instruments are not designed to collect intensity or bouts of activity. Regardless, these data serve as important reference values [25] for researchers and practitioners as well as families who are interested in collecting and comparing their own data using a more accessible research tool. The continued use and sharing of pedometer data is defensible as it represents a clear opportunity to bridge science, practice, and real life.

## Conclusions

In summary, the CANPLAY parent–child data indicate that children’s PA is related to that of their parents in distinct and quantifiable ways. Parents are a logical target for children’s PA interventions as changing a parent’s PA to a realistic target of 10,000 steps/day may increase their children’s PA behaviour. Interventions are warranted to evaluate the direction of the parent–child relationship, confirm the magnitude of influence, and illuminate mediating and moderating mechanisms by which both parents may have influence over their own children’s PA.

## Competing interests

The authors declare that they have no competing interests.

## Authors’ contributions

CLC and CC monitored original data collection. All authors conceived and designed the present analysis. CLC lead the analysis and results presentation with contributions from CC. CT-L and CLC lead interpretation of data with contributions from CC. CT-L and CLC led the writing however all authors critically reviewed and approved the final manuscript.
